# More Drugs and Fewer Strokes? Time Trends in CVD Medication and Incidence of Stroke With German Health Insurance Data

**DOI:** 10.1002/pds.70077

**Published:** 2025-01-07

**Authors:** Lieselotte Mond, Siegfried Geyer, Juliane Tetzlaff, Karin Weißenborn, Johanna Schneider, Jelena Epping

**Affiliations:** ^1^ Medical Sociology Unit Hannover Medical School Hanover Germany; ^2^ Department of Neurology Hannover Medical School Hanover Germany

**Keywords:** drugs, ecological correlation, preventive medication, routine data, stroke

## Abstract

**Background:**

Successful prevention of cardiovascular diseases (CVD) may reduce the burden of diseases. Preventive medication is an important measure to decrease the risks of cardiovascular events, in particular myocardial infarction and stroke. The aim of this study is to analyze the prevalence of CVD preventive medication in Germany over time with respect to sex and age and to compare it with the temporal development of strokes.

**Methods:**

The study is based on statutory health insurance claims data from the AOK Niedersachsen (AOKN) covering the years 2005–2018. The study population comprises all AOKN insured persons aged 18 years and older (*N* = 2 088 495). Age‐standardized time trends of the prevalence of CVD preventive medication and incidence of stroke were calculated for men and women in different age groups. After that, the relationship of both measures was examined in an ecological correlation.

**Results:**

We found a clear increase in medication prevalence over time. In 2018, about 35% of the total population and about 85% of those over 85 years of age received CVD preventive medication. At the same time, age‐standardized incidence rates of ischemic stroke were decreasing slightly. The ecological correlation showed a negative association between medication prevalence and stroke incidence especially in the higher age groups.

**Conclusion:**

High correlation coefficients indicate that higher medication prevalence could be linked to better population health. Further research is needed to draw conclusions about the effects of increasing medicalization, including adverse risks and side effects at the population level.

**Summary**

- In the insured population of a large statutory health insurance provider in Germany, age‐standardized prevalence rates of CVD preventive medication increased over time.
- In 2018, about 35% of the total population and about 85% of those over 85 years of age received CVD preventive medication.
- Simultaneously, age‐standardized incidence rates of ischemic stroke decreased slightly.
- An ecological correlation at a population level showed a negative association between medication prevalence and stroke incidence in the higher age groups.
- Further research is needed to draw conclusions about the effects of increasing medicalization, including adverse risks and side effects at the population level.

## 1 Background

Cardiovascular diseases (CVD) are a major cause of mortality and morbidity worldwide and in Germany [1], thus successful prevention may affect population health. Underperforming CVD prevention is even discussed as one reason for Germany's laggard position in life expectancy compared to other high‐income countries [2]. It is therefore essential to analyze CVD prevention measures in Germany and assess their impact on the health of the population. Preventive medication plays an important role in reducing the risk of cardiovascular events at the individual level, particularly in myocardial infarction and stroke [3]. To evaluate drug prescription as a preventive measure at the population level, monitoring and analysis of prescription data is essential. This study shows how the prevalence of prescriptions of CVD preventive medication within the population of a statutory health insurance provider in Germany developed over time and to what extent there is an association with the temporal development of strokes.

International studies suggest an increase in drug use, including CVD preventive medicine: A Danish registry study found a significant increase in the odds of treatment with ASA, RAS inhibitors, and statins between 2000 and 2008 in a cohort of individuals with first‐time indications for coronary angiography. In contrast, the odds of treatment with beta‐blockers decreased [4]. Another Danish study showed a marked increase from 1998 to 2018 in CVD preventive medicine in patients with abdominal aortic aneurysm [5].

Representative data from population surveys on the use of drugs in Germany were assessed since 1990 and show increases in drug user prevalence from about 60% in the early 1990s to more than 70% in 1998 [6] and nearly 75% between 2008 and 2011 [7]. Medication use was reported to be higher in women and to increase with age, although this age dependency was more pronounced among men than among women and gender differences thus decreased with increasing age [6]. A strong age dependency was found in later health surveys in Germany, as well [8].

For specific medication groups, the Pharmaceutical Prescription Report publishes drug use data based on routine data from statutory health insurance providers since 1985. For CVD‐preventive medication an overall increase in prescription numbers was recorded for the period 2011–2020, even though the prescription of some medication groups is declining (e.g., vitamin K antagonists, fibrates, human insulins) [9]. The same development for drug prescriptions relevant to coronary heart disease (CHD) was shown in the Health Atlas of the Scientific Institute of a large statutory health insurance fund (WIdO): Compared to the index year 2010 (100%), the amount of prescribed CHD‐relevant drugs was 70% in 2000 and more than 120% in 2020 [10].

In view of Germany's aging population, this increase in crude prescription numbers is not surprising. Unfortunately, neither the prescription numbers reported in the Pharmaceutical Prescription Report [9] nor those in the Health Atlas [10] provide any reference to the population size and changing age structure of the population over time. Findings on age‐standardized and sex‐specific differences in the temporal development of CVD preventive medication in Germany are lacking so far. However, the observation of time trends in drug prescriptions is important to better assess the preventive effects at the population level.

Previous studies based on German health insurance data showed decreasing stroke incidence between 2006 and 2014/2016 [11, 12] with a greater increase in stroke‐free life years in men than in women [12].

The aim of this study is to analyze the temporal development of prescriptions of CVD preventive medication within the insured population of the AOK Lower Saxony. Furthermore, the prescription of CVD preventive medication will be analyzed in association with ischemic stroke as one of the most serious outcomes of CVD. The research questions are as follows:

1. Are there increases in the prescription of CVD preventive medication over time?
2. Does the development of CVD preventive medication differ between men and women?
3. Does the development of CVD preventive medication differ between age groups?
4. How has the incidence of ischemic stroke in men and women evolved over time?
5. Is there an association between the development of the prevalence of CVD preventive medication and the occurrence of ischemic stroke at population level over time?

## 2 Methods

### 2.1 Data

Analyses were performed based on anonymized health insurance claims data of a large statutory health insurance, the Allgemeine Ortskrankenkasse Niedersachsen (AOKN) covering about 37% of the population of Lower Saxony, Germany [13]. All insured persons 18 years and older were included in the analyses, that covered the time period from 2005 to 2019. Each year, approximately 2 million individuals are included. In Germany, health insurance is mandatory. Up to a defined income threshold (about 66 000€ in 2019), individuals must have health insurance within a statutory health insurance scheme. The statutory health insurance covers a very wide range of health services including medication prescription, that is, no financial barriers exist for insured individuals to obtain the recommended medication.

### 2.2 Prevalence of Medication

In statutory health insurance claims data, the information on drug prescription is available on an ATC (“Anatomical Therapeutic Chemical”) code basis. Only prescriptions that were picked up at the pharmacy are registered in the data.

Based on the current literature, on the S3 guideline “Stroke” of the German Society of General Practice and Family Medicine [14] and in coordination with the Department of Neurology of Hannover Medical School, Germany, a complete list of CVD preventive medication was compiled. The focus was set on primary and secondary prevention of ischemic stroke. The following drug groups are included: antihypertensives, antiplatelet agents, oral anticoagulation (vitamin K antagonists, NOAKs), statins and fibrates, insulins and analogs, oral antidiabetics (ATC‐Codes of the groups of medications are displayed in Supporting Information: Table A).

To approximate the effective use of a medication, only individuals with at least two prescriptions within the same medication group, no more than 200 days apart within a year were counted as prevalent. To calculate prevalence rates, individuals meeting the mentioned prerequisites for at least one drug group from the defined list of CVD preventive medications were counted for each year. The number of insured persons for each year was determined as the denominator.

For the age standardization, the population insured in 2012 was defined as the standard population. To compare prevalence rates for men with those for women and to avoid the influence of different age structures between men and women, the 2012 standard population was not stratified by sex for age standardization. Analyses were performed both for the entire insured population stratified by sex and according to six age groups.

### 2.3 Stroke Incidence

In claims data of German health insurance, diseases are coded according to ICD‐10. For these analyses, ICD‐10 code I 63 (ischemic stroke) was considered, using the data from the inpatient sector.

In order to identify the first‐ever ischemic stroke event, and to distinguish between prevalent and incident cases in the data, lookback periods of 365 days were applied. Thus, individuals with a stroke in the first 365 days of the individual insurance period were not considered as incident and were excluded from the calculations. For some persons, their first stroke event happened in the fifth, for some others in the eighth year of their individual insurance period. Thus, all available time before we identified the first stroke within these health insurance data was used and 365 days were only a minimum lookback period.

The length of the lookback period was chosen based on earlier studies [12, 15].

The observation time of all insured persons calculated as person‐years builds the denominator. As the first insurance year acts as a lookback period, it was excluded from the observation period. Once individuals have suffered a stroke they are no longer observed. In addition, leaving the insurance (e.g., by death) terminates the observation.

Age standardization was performed analogously to the age standardization of the medication prevalence rates. The results are reported for the entire insurance population as well as stratified by sex and six age groups.

### 2.4 Correlations

The first choice in an analysis on relationships between medication rates and diseases (in the present case ischemic stroke) is to calculate associations from the same individual level‐dataset. However, it is not conceivable that medication is prescribed in the absence of risk factors, in this case high blood pressure, hyperlipidemia, stenoses of the brain supplying arteries or the peripheral arteries, overweight or adiposity, which makes analyses based on individual data very challenging. In order to have a proper design for estimating effects by means of regression analyses, it is necessary to have (1) cases with medication in the presence of a diagnosis/risk factor potentially leading to an outcome, and (2) having a diagnosis/risk factor without medication potentially leading to an outcome. However, the latter case would hardly occur, because physicians will hardly leave diagnosed risk factors untreated, and there will be no medication without the presence of risk factors. In order to overcome this problem, we decided to pursue an ecological approach by drawing aggregated medication data from our health insurance dataset and the ischemic stroke rates from aggregated disease data in parallel. With this approach, this study focusses on time trends at a population level and not on the effectiveness of cardiovascular medication.

Correlations were calculated at population level in terms of ecological correlations. Stroke incidence rates of 1 year were correlated with CVD preventive drug prevalence rates of the previous year. This shift was implemented after consultations with clinical experts since in this manner it could be accounted for the rather long‐term onset of action of the drugs. Due to very low stroke incidence rates for age groups under 65 years of age, only correlations for persons aged 65 years and older are shown in this article. Correlations for the younger age groups can be seen in Supporting Information (Figure A).

## 3 Results

Descriptive statistics of the study population are shown in Table 1.

**TABLE 1:** Descriptive statistics of the study population: Number of persons, cases with CVD‐preventive medication and incident stroke cases by year and sex. For incident stroke cases, 2005 is pre‐observation time only.

Year	*N*		Number of cases with CVD‐preventive medication		Number of incident stroke cases		Average age in years	
	Men	Women	Men	Women	Men	Women	AM	SD
2005	900 447	1 041 207	233 921	349 707			51.1	19.6
2006	894 671	1 030 193	246 970	364 115	2806	3627	51.1	19.7
2007	888 334	1 018 323	253 721	367 858	3021	3667	51.2	19.8
2008	874 670	999 737	260 546	372 300	2921	3764	51.4	19.9
2009	875 460	993 016	267 367	374 806	2932	3499	51.3	19.9
2010	1 012 276	1 103 029	307 442	408 671	2814	3442	50.9	19.8
2011	1 020 403	1 104 166	315 232	413 388	3270	3648	50.8	19.9
2012	1 025 852	1 101 935	321 229	415 056	3328	3713	50.8	19.9
2013	1 026 331	1 094 597	325 501	414 308	3274	3722	50.8	19.9
2014	1 032 010	1 092 367	328 641	413 075	3241	3640	50.8	19.9
2015	1 049 149	1 101 921	333 592	413 093	3348	3575	50.6	19.9
2016	1 093 557	1 135 496	342 280	417 096	3316	3695	50.2	19.9
2017	1 142 647	1 180 776	351 522	422 074	3426	3515	49.7	19.8
2018	1 184 309	1 222 052	360 752	427 759	3415	3394	49.4	19.7

### 3.1 Development of Medication Prevalence

**FIGURE 1:**
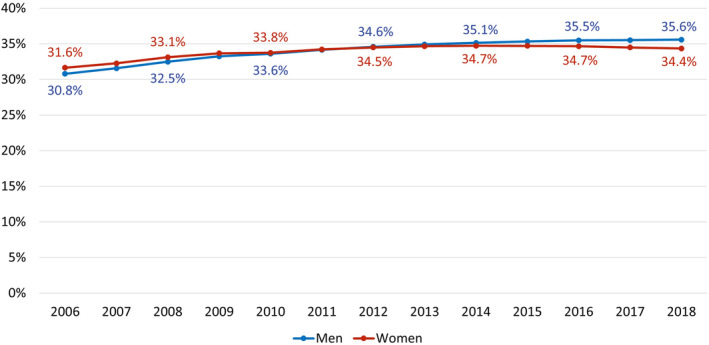
Age‐standardized prevalence rates of CVD preventive medication for men and women over time aged 18 years and older.

Over the whole observation period, more than 30% of all adult insured persons of the AOK Lower Saxony are medicated with at least one drug from the defined list of CVD preventive medication (Figure 1). Over time, a clear increase in medication prevalence can be seen at a different pace for men and women. In 2006, the age‐standardized medication prevalence of men is 0.8 percentage points lower than the medication prevalence of women (30.8% vs. 31.6%). At about the midpoint of the observation period, rates for women and men are equal at 34.2% in 2011, and at the end of the observation period, prevalence for men is higher than prevalence for women (35.6% vs. 34.4% in 2018). The relative increase in medication prevalence over time amounts to 15.6% for men and 8.6% for women.

**FIGURE 2:**
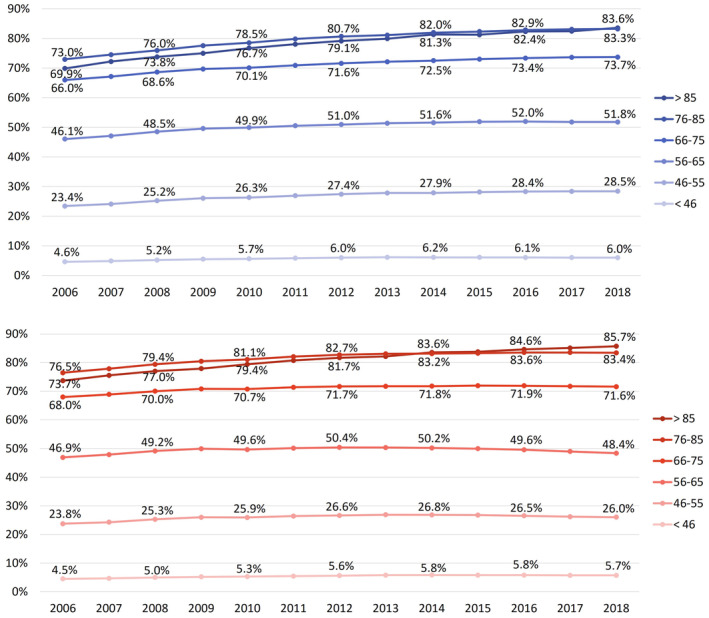
Age‐standardized prevalence rates of CDV preventive medication stratified by age groups for men (blue) and women (red) aged 18 years and older.

Stratified by age groups, the expected pattern emerged (Figure 2): the higher the age, the higher the CVD medication prevalence rate. An exception are the prevalence rates of the oldest insured persons (over 85 years of age), which are below those of the 76–85 year‐olds until around 2013/2014, to then overtake and show the highest prevalence rates in 2018.

The previously described increase in the prevalence of CVD preventive medication affects all age groups, in both men and women. In absolute terms, the largest increase from 2006 to 2018 is found in men over 85 years with 14% points (from 69.9% to 83.6%), followed by women over 85 years with 12% points (from 73.7% to 85.7%). The relative increase, on the other hand, is greatest in the youngest age group, though the absolute prevalence levels are quite low. It amounts to 30% among men under age 46 (from 4.6% to 6.0%) and 26% among women in the same age group (from 4.5% to 5.7%).

### 3.2 Development of Ischemic Stroke Incidence

**FIGURE 3:**
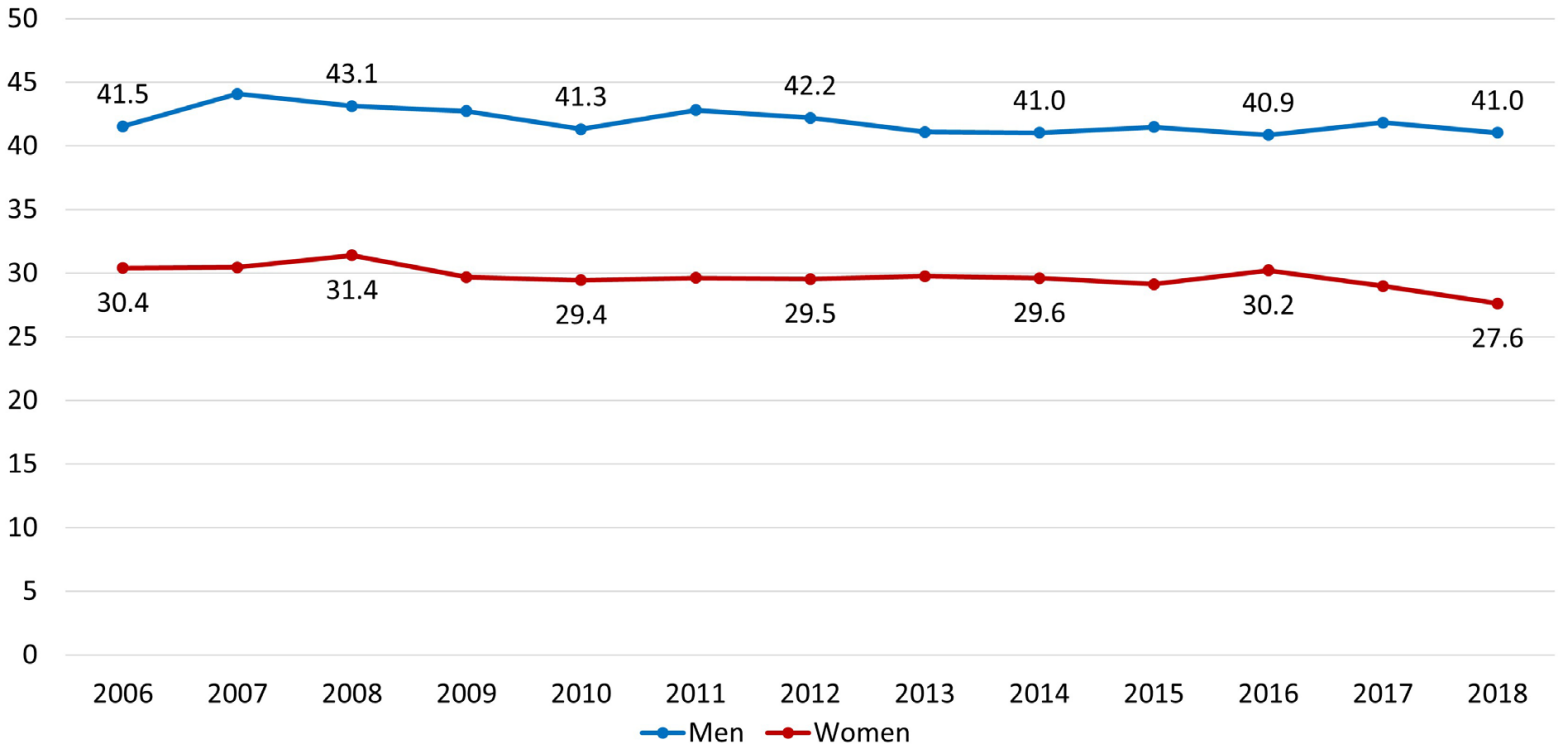
Age‐standardized incidence rates of ischemic stroke (cases per 10 000 person‐years) for men and women aged 18 years and older over time.

During the entire observation period, age‐standardized stroke incidence rates for men were higher than those of women (about 43 vs. 30 events per 10 000 person‐years). From 2006 to 2018, incidence rates were stable or slightly decreasing. Considering the consistency of the trend over time, this downward trend was slightly more apparent for men than for women (Figure 3).

**FIGURE 4:**
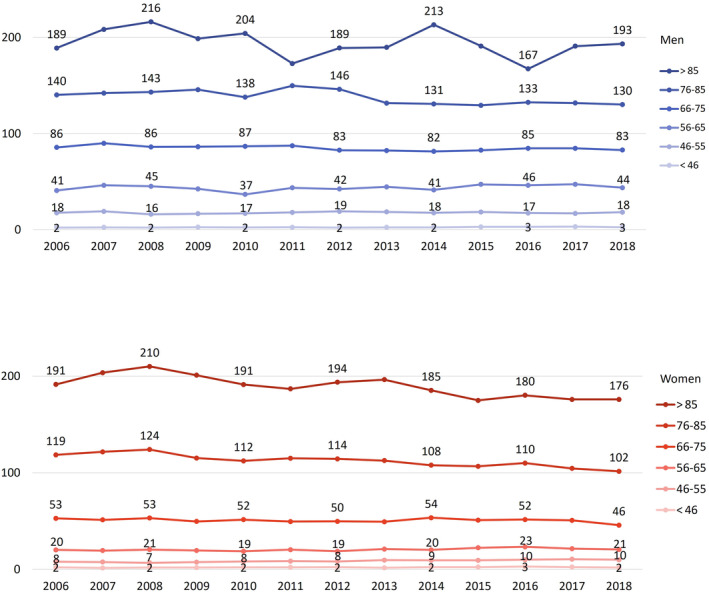
Age‐standardized incidence rates of ischemic stroke (cases per 10 000 person‐years) stratified by age groups for men (blue) and women (red) aged 18 years and older.

Regarding the age groups (Figure 4), the downward trend in stroke incidence is more apparent for the elderly. In women, the two oldest age groups (76–85 and > 85 years) show a downward trend (after an initial increase in stroke incidence from 2006 to 2008) from 124 resp. 210 cases per 10 000 person‐years in 2008 to 102 resp. 176 cases in 2018. In men, age‐standardized stroke incidence rates show similar tendencies as in women though in the two oldest age groups there is more variation (despite crude case numbers e.g., for the oldest men between 194 and 354 cases per year). The incidence rates of the youngest three age groups remains quite stable. Overall, stroke incidence rates in the younger age groups are very low.

### 3.3 Ecological Correlations

**FIGURE 5:**
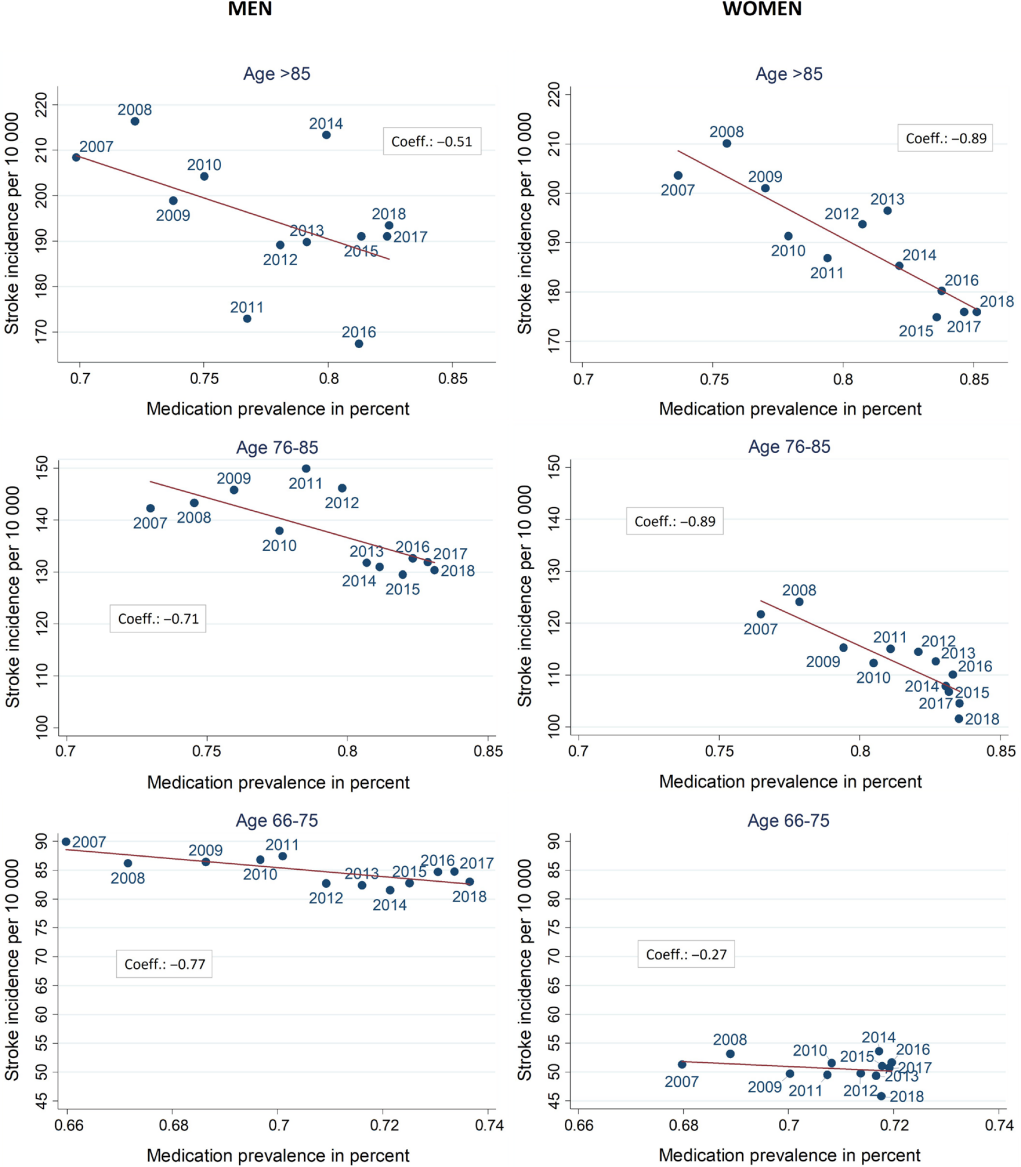
Scatterplots and correlation coefficients (Coeff.) for age‐standardized prevalence rates of CVD preventive medication and age‐standardized incidence rates of ischemic stroke, stratified by gender and age group. The annual figures refer to the stroke incidence rates (and are correlated with prevalence rates of medication from the previous year).

Due to very low stroke incidence rates in lower age groups, ecological correlations between the CVD preventive medication prevalence and ischemic stroke incidence were calculated only for individuals older than 65 years of age, stratified for three age groups. As seen in Figure 5, negative correlations occur over time for men and women indicating that increasing CVD medication prevalence in the year *X* correlates with decreasing ischemic stroke incidence in the year *X* + 1 over time. For men and women aged 76 years and older, the correlation is moderate to strong (−0.51 to −0.89). In the age group 66–75 years, the correlation coefficients differ substantially between men and women: for men, a correlation coefficient of −0.77 shows a strong association between increase in medication prevalence and decrease in ischemic stroke incidence. For women in this age group, the correlation is weak (−0.27).

## 4 Discussion

To our best knowledge, this is the first study to examine the development of CVD‐preventive medication over time by analyzing age‐standardized and sex‐specific prevalence rates. It has been shown that CVD‐preventive medication is widespread and that prevalence rates were rising over time, especially in men. In particular, this applies to men in the highest age groups. Furthermore, this is the first study to link the temporal development of medication prevalence rates with the temporal development of stroke incidence rates at a population‐level.

### 4.1 Medication Prevalence

In 1985, medication use was referred to by Rose as a “high‐risk strategy” (a preventive measure for a small number of individuals with an extraordinary risk profile) compared to the lifestyle population approach [16]. Thirty‐three years later, Sniderman et al. noted that things have changed: medication use has become the most frequent way of primary prevention of CVD [17]. Our results confirm this. Between 2006 and 2018 more than one third of our adult insurance population were treated with CVD‐preventive medication. In the United States, the use of CVD‐preventive medication might be even higher: For statins, a study by Pencina et al. reported more than 40% of the US adult population in 2005–2010 to have a respective indication. A direct comparison is still difficult: Pencina et al. refer to only one group of medication and to the number of persons having an indication [18], while in our study we counted several groups of CVD‐preventive medication together and looked at the number of persons collecting (regularly) their prescribed medication from the pharmacy.

The increase in age‐standardized prevalence of CVD preventive medicine over time in our study is in line with international studies [4, 5] and shows that the increasing prescriptions in Germany [9] are not solely due to demographic developments. One reason for this may be the increasing evidence for the effectiveness of the drugs, especially statins, and the lowered threshold for indication in this context, as found in the US American guidelines for lowering blood pressure [19]. Furthermore, there have also been changes in the European guidelines during the observation period: here, the therapeutic target blood pressure values have been lowered, which could also have influenced the use of CVD preventive medication [20]. It can be assumed that the United States and European guidelines also influence prescription behavior of German physicians.

Another reason for the increase in drug prevalence may be an increase in CVD‐related risk factors over time. A Swedish registry study shows increasing prevalence of hypertension from 1969 to 2010 for 18‐year‐olds, especially for men [21]. Another study, based on the US National Health and Nutrition Examination Survey 1999–2004, finds similar results for young adults (18–39 years) [22]. In contrast to this, several studies show decreasing hypertension prevalence rates in Germany [23, 24, 25], but this is considered to be the result of the increasing medication. For Type 2 diabetes, Muschik et al. report an expansion among insured persons of the AOK Lower Saxony during the observation period 2006 to 2013 with a decrease in the age of onset over time in the youngest age group (18–39 years) [26] and obesity rates in Germany are increasing, too [27].

The development of medication prevalence over time shows an upward and ultimately even overtaking effect in men compared with women. On the one hand, this could be due to the fact that the risk factors that can be influenced by medication have increased more in men than in women: At least for hypertension, such trends are evident [21, 22]. On the other hand, it may indicate that men are converging with women in their health‐conscious behavior and may therefore show increasing utilization of health care services. Indications that men are catching up in terms of health‐related behavior include stronger declines in smoking rates among men compared to women [28] and stronger increases in life expectancy [29]. Furthermore, sensitization of physicians for the preventive treatment of men might have increased.

### 4.2 Ischemic Stroke Incidences

An analysis of the Global Burden of Disease Study shows a relative decline of 10% (95% CI: −8% to −12%) in age‐standardized incidences of ischemic stroke from 1990 to 2019 worldwide, with a 2019 incidence of 94.51 (81.9 to 110.76) per 100 000 persons [30]. For Germany, a decrease from 176.31 (151.01 to 204.40) to 141.66 (119.49 to 164.84) is reported for the time period 1990 till 2010 as well [31]. These results were replicated for the time period 2006–2014 based on German health insurance claims data [11]. Another study showed decreasing stroke incidence in men and women and increasing life years free of stroke in men [12]. These reports highlight the downward trend in stroke incidence, slightly more apparent in men than in women, that is visible in our results as well.

### 4.3 Correlations

We found strong to moderate correlation between prevalence of CVD‐preventive medication and stroke incidence in the higher age groups. High correlation coefficients only occur if both indicators show a development over time. In the age group 66–75, stroke incidence rates as well as prevalence of CVD‐preventive medication developed more in men than in women. Therefore, the correlation was stronger in men than in women, indicating that men might have been underserved and are catching up over time.

The assumption that increasing medicalization might be the reason for decreasing stroke incidence is also shared by studies from other countries [32] and is supported by the evidenced effectiveness of CVD‐preventive medication on the individual level [33, 34, 35]. Further research is needed to detect adverse risks and side effects of increasing medication prevalence at the population level.

### 4.4 Strengths and Limitations

The great strength of routine data, apart from the fact that they do not have to be collected in a resource‐intensive way, lies on the one hand in the high number of cases and on the other hand in the absence of strong selection effects as they occur in surveys [36]. The state of Lower Saxony has rural regions as well as small, medium and large cities, and the population of insured persons of the AOK Lower Saxony is representative of the population of the Federal Republic of Germany in terms of age and sex [37]. Due to the historical development of statutory health insurance in Germany, persons with lower socioeconomic status are overrepresented in these health insurance data [37]. With regard to medication prescriptions (defined daily doses and sales per insured person), the state of Lower Saxony is in the midfield in a national comparison [38].

There is no uniform definition of CVD preventive medicine. In particular, the inclusion of antidiabetic drugs is debatable, as their effect on the cardiovascular system is indirect. In the European guidelines for cardiovascular prevention, diabetes therapy is recommended to reduce cardiovascular risk [3]. Because diabetes is an important risk factor for cardiovascular events such as ischemic stroke [32], its medication was added to CVD preventive medicine in this analysis. The drugs considered are all capable of preventing strokes but have a wide range of indications at the same time, which means that the prevention of ischemic stroke is not the only effect the drugs might have.

The data include all drug prescriptions picked up at a pharmacy. This does not necessarily mean that the drugs were also taken [39]. To reduce the gap between medication pick‐up and intake in our results, and to be able to assume that the medication had an effect, the minimum requirement of at least two prescriptions no more than 200 days apart was set. Furthermore, it is not visible in the data to what extent persons take medication without prescription or deviating from the prescription (self‐medication) [8]. Since the drugs considered here, with the exception of ASA, are prescription drugs, self‐medication is not considered to have a serious limiting effect on the study results.

As in all ecological studies, there is a risk of the ecological fallacy because medication prevalence rates and strokes are not associated at the individual level [40]. The presence of a statistical correlation may therefore not be interpreted as evidence of a causal relationship. Population prevention approaches, such as tobacco prevention policies, improving working conditions, and so forth, may as well have contributed to the decline in stroke incidence.

In principle, routine data already represent an important data basis in pharmaco‐epidemiology and are increasingly used by decision makers in the public health sector [41].

### 4.5 Conclusion

Age‐standardized prevalences of CVD preventive medication are increasing in all age groups, with greater increases in men than in women and older age groups, suggesting that men are catching up in terms of increased use of the health services. In 2018, about 35% of the total population and about 85% of those over 85 years of age were treated with CVD preventive medication.

At the same time, age‐standardized incidence rates of ischemic stroke, primarily in the older age groups, are decreasing slightly over time.

High correlation coefficients indicate that higher medication prevalence could be linked to better population health. Further research is needed to draw conclusions about the effects of increasing medicalization at the population level, including shedding light on adverse risks and side effects at the population level.

## Author Contributions

J.E. developed the idea and research questions of the study. L.M. analyzed the data and wrote the first draft of the manuscript. S.G. and J.E. were major contributors to the final manuscript. K.W. and J.S. contributed to the conception and discussion of the study and reviewed the work critically. All authors read, edited, and approved the final version of the manuscript.

## Ethics Statement

The analyses conducted in this study utilize a pre‐existing claims dataset, originally collected as part of routine administrative processes by a statutory health insurance provider. The use of such data is governed by German law, specifically the German Civil Code (“Bürgerliches Gesetzbuch”). Permission to use the data for scientific research was granted by the data protection officer of the Local Statutory Health Insurance of Lower Saxony (AOK Niedersachsen, Germany). In accordance with these legal requirements, ethical approval and consent to participate were not necessary for this study. The data were fully anonymized before access was granted. Furthermore, we affirm that all methods were carried out in compliance with relevant guidelines and regulations.

## Conflicts of Interest

The authors declare no conflicts of interest.

## Supporting information


Table A.
Figure A.

## Data Availability

The data analyzed in this study cannot be made publicly available due to protection of data privacy of the insured individuals by the AOK Niedersachsen (AOKN‐Statutory Local Health Insurance of Lower Saxony). The data underlying this study belong to the AOKN. Researchers interested in the data supporting the conclusions of this article can send data access requests to the AOK Niedersachsen using the following e‐mail address: aok.service@nds.aok.de.
