# Supplementary material for: More Drugs and Fewer Strokes? Time Trends in CVD Medication and Incidence of Stroke With German Health Insurance Data
Source: Pharmacoepidemiol Drug Saf. 2025 Jan 7;34(1):e70077. doi: 10.1002/pds.70077 (PMC11706669; doi:10.1002/pds.70077)
Supplement: Supplementary file 1 — Table A. Figure A. [file PDS-34-e70077-s001.docx]

**Supplement:** More drugs and fewer strokes? Analysis of Time Trends in CVD Preventive Medication and Incidence of Ischemic Stroke Based on German Health Insurance Claims Data
*Mond, Lieselotte; Geyer, Siegfried; Tetzlaff, Juliane; Weißenborn, Karin; Schneider, Johanna; Epping, Jelena*

Table A: Included Mediacation groups

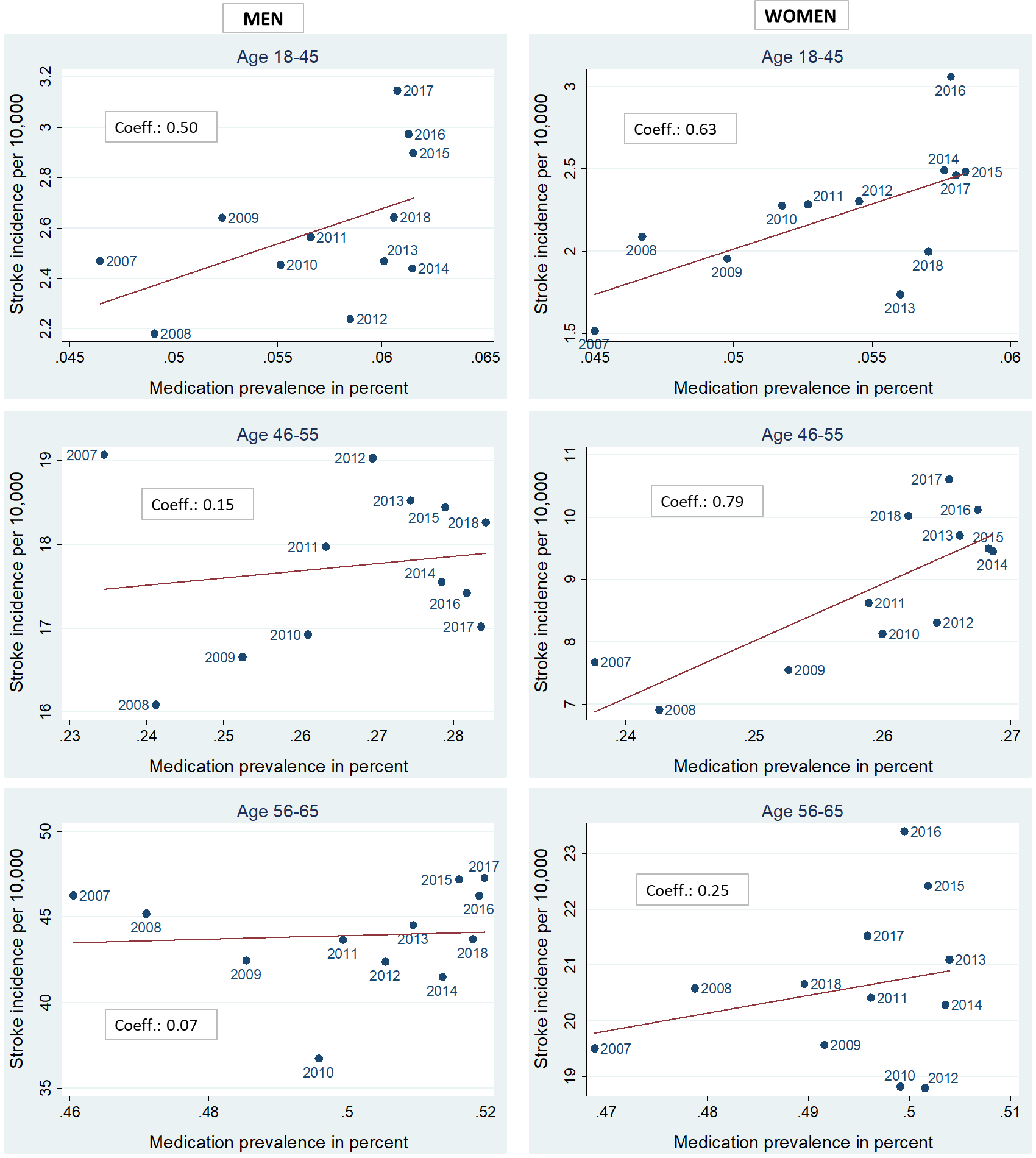


Figure A: Scatterplots and Correlation Coefficients (Coeff.) for age-standardized prevalence rates of CVD preventive medication and age-standardized incidence rates of ischemic stroke, stratified by gender and age group. The annual figures refer to the stroke incidence rates (and are correlated with prevalence rates of medication from the previous year).
